# Single Nucleotide Polymorphisms in Noncoding Regions of *Rad51C* Do Not Change the Risk of Unselected Breast Cancer but They Modulate the Level of Oxidative Stress and the DNA Damage Characteristics: A Case-Control Study

**DOI:** 10.1371/journal.pone.0110696

**Published:** 2014-10-24

**Authors:** Peter Gresner, Jolanta Gromadzinska, Ewa Jablonska, Maciej Stepnik, Oscar Zambrano Quispe, Ewa Twardowska, Wojciech Wasowicz

**Affiliations:** 1 Department of Toxicology and Carcinogenesis, Nofer Institute of Occupational Medicine, Lodz, Poland; 2 Department of Oncology, Herlev Hospital, Herlev, Denmark; Northwestern University Feinberg School of Medicine, United States of America

## Abstract

Deleterious and missense mutations of *RAD51C* have recently been suggested to modulate the individual susceptibility to hereditary breast and ovarian cancer and unselected ovarian cancer, but not unselected breast cancer (BrC). We enrolled 132 unselected BrC females and 189 cancer-free female subjects to investigate whether common single nucleotide polymorphisms (SNPs) in non-coding regions of *RAD51C* modulate the risk of BrC, and whether they affect the level of oxidative stress and the extent/characteristics of DNA damage. Neither SNPs nor reconstructed haplotypes were found to significantly affect the unselected BrC risk. Contrary to this, carriers of rs12946522, rs16943176, rs12946397 and rs17222691 rare-alleles were found to present significantly increased level of blood plasma TBARS compared to respective wild-type homozygotes (p<0.05). Furthermore, these carriers showed significantly decreased fraction of oxidatively generated DNA damage (34% of total damaged DNA) in favor of DNA strand breakage, with no effect on total DNA damage, unlike respective wild-types, among which more evenly distributed proportions between oxidatively damaged DNA (48% of total DNA damage) and DNA strand breakage was found (p<0.0005 for the difference). Such effects were found among both the BrC cases and healthy subjects, indicating that they cannot be assumed as causal factors contributing to BrC development.

## Introduction

Breast cancer (BrC) is the most common malignancy among women, with nearly 1.4 million new BrC cases causing nearly 460,000 deaths per year worldwide [1]. Although vast majority of BrC cases are sporadic, approximately 5–10% of them are considered familial, occurring at unusually young ages in members of families with strong history of hereditary breast and/or ovarian cancer (HBOC) [2]. Studies have shown that familial BrC is likely a polygenic disease caused by mutations in several high-, moderate- and low-penetrance susceptibility genes. Genes like *BRCA1*, *BRCA2*, *TP53* and *PTEN* are well-known high-penetrance susceptibility genes, although nowadays it is assumed, that they account for roughly some 20% of all hereditary breast cancer cases [3]–[5]. Therefore, the ongoing quest to identify additional BrC-susceptibility genes resulted in identification of several moderate-penetrance BrC-susceptibilty genes, the majority of which are somewhat related to *Brca1*/*Brca2*-mediated pathways. These include *CHEK2*, *ATM*, *BRIP1*, *PALB2* and recently also *RAD51C*, all of which are involved in various steps of DNA recombination repair [5]–[7]. An approximately 2-fold relative risk increase among carriers of heterozygous mutations in these genes has been implied [8].

Members of the *RAD51* gene family are often found among genes tested as possible BrC susceptibility genes. This family consists of *RAD51*, a key player in the homologous recombination (HR) double-strand breaks (DSBs) DNA damage response pathway, and its five paralogs: *RAD51B*, *RAD51C*, *RAD51D*, *XRCC2* and *XRCC3*. Protein products of these five genes interact with each other to create hetero-tetrameric and hetero-dimeric complexes crucial for the HR machinery [9]. Out of all *RAD51* paralogs, protein product of *RAD51C* (*RAD51* homolog C, *S. cervisiae*; 17q25.1) seems to play a prominent role as it is found in both the above mentioned complexes. Indeed, RAD51C localizes to the sites of DNA DSBs in early stage of HR, which is thought to be a prerequisite for RAD51/DNA nucleoprotein filament assembly, a key event in the whole HR reaction [10]. Other identified functions of this protein include facilitating the migration and resolution of Holliday junctions in late stages of HR [11], repair of interstrand cross-links [12], distinct functions related to DNA damage response and checkpoint activation [10] and a function of tumor suppressor and cancer susceptibility gene [5], [13]–[15]. It has also been shown, that HR complexes involving *RAD51C* protein product are engaged in repair of stalled/collapsed replication forks induced by single strand breaks (SSBs) of DNA [16]. Interestingly, a recent study has proposed that RAD51C (together with other RAD51 paralogs) may play a role in protection of mitochondrial genome against oxidative damage as well, as mitochondrial level of this protein was found to increase with oxidative stress and its depletion leads to dramatic decrease of mtDNA copy number [17].

Meindl et al. identified deleterious frame-shift, splice-site and missense mutations in *RAD51C* to associate with HBOC families [13] and this observation was further confirmed by other studies including those on unselected ovarian cancer (OC) cases [14], [18]–[22]. Despite the discussion on possible involvement of *RAD51C* genotyping in the routine clinical testing [18], there is still a considerable amount of studies which failed to find any association between *RAD51C* mutations and HBOC [15], [23]–[27], a fact, which is usually explained by very rare occurrence of these mutations. It is, however, intriguing that while all these mutations were associated with HBOC or OC only families, none of the above cited studies identified *RAD51C* mutations associated with BrC only families. This somehow indicates, that *RAD51C* is a susceptibility gene for HBOC and OC, but not for BrC and other cancers [28].

As all *RAD51* paralogs are generally considered conservative with the frequency of missense mutations being very low, we recently focused on single nucleotide polymorphisms (SNPs) occurring in promoter, 5′ untranslated region (5′UTR) of exon 1 and intron 1 of these genes and found that genetic variability of *XRCC3* and *RAD51* may be of relevance with respect to head and neck cancer (HNC) [29]. In the case of *RAD51C*, its association with BrC is generally sought in terms of its missense mutations in coding regions, while the intronic/promoter variability is rather underestimated. Interestingly, our recent study revealed that non-coding SNPs spanning from *RAD51C* promoter to its intron 1 form a linkage-disequilibrium (LD) block significantly associated with HNC risk [30]. Therefore, here we present a study, in which we employed a case-control setup to find out whether common SNPs occurring in non-coding regions (promoter, 5′UTR of exon 1, intron 1) of *RAD51C* may influence the risk of BrC. To this purpose, both single-site and haplotype analyses were performed. Moreover, in order to inspect the possible role of *RAD51C* in BrC more profoundly, we examined whether such variability of *RAD51C* affects the level of oxidative stress, and the extent of DNA strand breakage and/or oxidatively generated DNA damage, and if so, whether such effect may be associated with the development of BrC. In further we show that even though the variability in noncoding regions of *RAD51C* may not alter the risk of unselected BrC, it may be involved in modulation of the oxidative stress and may determine the characteristics of resulting DNA damage.

## Material and Methods

### Reagents

Diethylpyrocarbonate-treated water used for genotyping of *Rad51C* was obtained from Promega (Madison, WI, USA). Tetrasodium and disodium salts of ethylenediamine tetraacetic acid (Na_2_EDTA, Na_4_EDTA), thiobarbituric acid (TBA), acetic acid, 1-butanol, Tris base, type VII and type I agarose, Triton X-100, RPMI-1640 medium and 4′6-diamidino-2-phenylindole dyhydrochloride (DAPI) were purchased from Sigma-Aldrich (St. Louis, MO, USA), Phosphate buffered saline (PBS) and hydrochloric acid were from Polish Chemicals (Gliwice, Poland), while 1,1,3,3-tetraaethoxy-propan was obtained from Fluka (Buchs, Switzerland).

### Subjects

All breast cancer subjects enrolled in the study were of European descent and residents of Lodz district in Poland. The study involved 132 female patients aged 36–86 years (median age at the time of diagnosis 57 years; interquartile range (IQR): 15 years) hospitalized at the Department of Oncology, Memorial Copernicus Hospital in Lodz, Poland between February 2007 and May 2008 with diagnosis of BrC confirmed histopathologically by two independent histopathologists. Only female patients with primary breast cancer tumor without metastases and without any history of previous anti-cancer treatment, undergoing curative resection therapy or chemotherapy were eligible for the study. The control group consisted of 189 healthy cancer-free volunteer females of European descent who agreed to undergo examinations, aged 35 – 54 years (median age at the time of examination 43 years; IQR: 6 years). All control subjects were also residents of Lodz district in Poland.

Additional information on tobacco-smoking habits was collected for both controls and BrC cases and the individual's lifetime tobacco consumption was expressed by means of the pack-years (i.e. the number of cigarette packs smoked per day times the number of years as a smoker).

For the purposes of DNA damage assays, a subset of 40 controls randomly selected from the group of all control females enrolled in the study was created. To achieve this goal, a method of age-stratified randomization was employed in order to ensure that age distribution in the control subset match the one in the group of BrC females. Following randomization, the matching of SNP distribution between control subset and the whole control group was also checked.

Prior to experiments, written and informed consent for participation in this study was obtained from each subject enrolled. The study was performed under the guidelines of the Helsinki Declaration for human research and was approved by the Bioethics Committee in the Nofer Institute of Occupational Medicine (resolution no. 5/2007). Characteristics of the breast cancer group, the control group as well as the subset of controls used for DNA damage assays are summarized in Table 1.

**Table 1:** Characteristics of the groups of subjects involved in the study.

Feature	Cancer cases	Control subjects	DNA damage assay control subset a
Total number	132	189	40
Age [years]	57 [50–65] b	43 [40]–[46]	43 [40]–[48]
Smoking status [never/ever]	55/63 (47%/53%) c	120/68 (64%/36%)	24/16 (60%/40%)
Pack-years	11.3 [6.0–20.0] d ^,^ e	2.0 [1.0–4.6]	1.0 [0.8–4.6]
Tumor staging f			
*T [1/2/3/4/x]*	44/45/1/6/1	-	-
*N [0/1/2/3/x]*	44/24/10/2/20	-	-
Tumor grade			
*G [1/2/3/x]*	7/37/40/13	-	-

Numerical data for age and pack-years presented as median [interquartile range].

a DNA damage assay control subset consisted of 40 subjects randomly selected from the whole control group by means of the age-stratified randomization;

b p <<0.001 cancer cases vs. controls, Mann-Whitney *U* test;

c p <0.005, cancer cases vs. controls; two-sided mid-P test;

d p <<0.0001, cancer cases vs. controls, Mann-Whitney *U* test;

e p <0.001, cancer cases vs. DNA damage assay control subset, Kruskal-Wallis *H* test *post-hoc* analysis;

f Tumor staging classification: T – describes the size of the primary tumor and its invasiveness, N – describes the regional lymph nodes involved, *x –*data not available.

### Blood collection

A sample of peripheral blood was collected from each subject involved in the study by venipuncture into tubes containing either EDTA or heparin as anticoagulants. Heparinized blood samples were then centrifuged (10 min, 1500×g, 4°C) to obtain plasma and used for assessment of the level of oxidative stress. Whole blood samples collected on EDTA were further used in genotyping and DNA damage assays.

### DNA isolation

Genomic DNA was isolated from peripheral blood leukocytes using the QIAamp DNA Blood Mini Kit (Qiagen, Germany) according to the manufacturer's instruction. The RNA contamination was removed by digestion with 1 mg/ml RNase A (Qiagen, Hilden, Germany) and obtained DNA was further quantified and the protein content and DNA purity was checked using an Eppendorf BioPhotometer (Eppendorf, Hamburg, Germany) instrument. Samples were stored at -80°C until further processing.

### Genotyping

In this study, we focused on single nucleotide polymorphisms (SNPs) occurring predominantly in non-coding region of *RAD51C*, as it is assumed that these SNPs may be involved in gene expression regulation. We involved seven SNPs localized in gene's promoter, 5′UTR of exon 1 and intron 1. Only those SNPs the minor allele frequency (MAF) of which in the Caucasian population exceeded 10% (according to data contained in dbSNP database [31]) were selected for the study. Possible involvement of selected SNPs in regulation of *RAD51C* expression was tested by is-rSNP algorithm [32], an *in-silico* method for prediction whether an SNP can be considered as regulatory (i.e. disrupting the transcription factor binding). Exemplary results of such prediction for selected well-known nuclear transcription factors together with other detailed information on SNPs analyzed in this study are provided in Table 2.

**Table 2:** Resume of *RAD51C* SNPs analyzed in the study.

					Disruption of binding of selected nuclear transcription factors (*p-value*)				
rs-code	New name	Other names	SNP position	MAF	NF-κB	Pax-5	Bcl-6	c-Myb	p53
rs302874	c.-2009C>T	g.3034C>T	5′ near gene (promoter)	T: 0.42	-	-	-	-	0.0052
rs12946522	c.-1902T>G	g.3141T>G	5′ near gene (promoter)	G: 0.17	0.0023	-	0.0028	-	0.0076
rs302873	c.-524C>G	g.4519C>G	5′ near gene (promoter)	G: 0.41	0.0448	0.0698	-	0.0002	-
rs16943176	c.-118G>A	g.4925G>A	5′ near gene (promoter)	A: 0.20	0.0133	0.0385	0.0391	0.0281	0.0490
rs12946397	c.-26C>T	g.5017C>T	exon 1 (5′UTR)	T: 0.21	0.0117	0.0642	0.0069	-	0.0196
rs17222691	c.145+947C>T	g.6134C>T	intron 1	T: 0.20	0.0461	0.0051	0.0257	0.0131	0.0034
rs28363302	c.146-706delC	g.6624delC	intron 1	del: 0.18	*NA*	*NA*	*NA*	*NA*	*NA*

SNP nomenclature, localization and minor allele frequencies (MAF) are provided according to data obtained from the dbSNP database [31]. Additionally, the levels of significance (p-values) for observing the change in binding affinity between the *RAD51C* promoter and several well-known transcription factors caused by SNPs investigated in our study, as inferred by the is-rSNP algorithm [32] against the Transfac database [48] are also provided. Dash indicates that the given SNP is unlikely to affect the binding of given transcription factor. *NA –* not applicable.

All SNPs involved in this study were genotyped in DNA isolated from the whole blood samples by means of the real-time PCR technique using either custom or pre-designed commercially available TaqMan SNP Genotyping Assays (Life Technologies, Carlsbad, CA, USA) according to manufacturer's instructions. Genotyping was performed on BioRad's iQ5 iCycler Multicolor Real Time PCR Detection System (BioRad, Hercules, CA, USA) in 20-μl aliquots containing 16 ng DNA. The cycling conditions, preceded by polymerase activation (95°C, 10 min) consisted of 50 cycles involving DNA denaturation at 95°C for 15 s followed by a combined annealing-elongation step at 61.5°C for 1 min, during which the fluorescence signal was measured. The genotype recognition of analyzed subjects was performed automatically using the BioRad's iQ5 Optical System Software ver. 2.0.

Ten per cent of obtained results were verified by repeated genotyping using the same technique.

### Determination of thiobarbituric acid-reactive species in blood plasma

An individual's level of oxidative stress was evaluated based on the amount of thiobarbituric acid-reactive species (TBARS) assayed in the plasma obtained from the heparinized peripheral blood sample. The concentration of TBARS was determined using the method previously described by Wasowicz et al. [33], based on the reaction of 2-thiobarbituric acid (TBA) with malondialdehyde, which is a naturally occurring side-product of lipid peroxidation. Briefly, a 50-μl aliquot of blood plasma was mixed with 1 ml deionized water, 25 µl of 5 M HCl and 1 ml of 12.2% (v/v) acetic acid containing 29 mM TBA. The mixtures were incubated for 1 h at 95–100°C and subsequently cooled on ice. After addition of 3.5 ml of 1-butanol, the samples were vortexed thoroughly for 2 min and centrifuged at 2880×g, room temperature for 10 min. Finally, the concentration of TBARS was assessed by measuring the fluorescence of the butanol extract at wavelength of 547 nm following excitation at 525 nm on using a PerkinElmer LS50-B instrument (PerkinElmer, Shelton, CT, USA). The concentration of TBARS in blood plasma was expressed in μM. A dilution series of 1,1,3,3-tetraaethoxypropane was used for standard curve preparation.

### DNA damage assays

DNA damage including the single-strand breaks (SSBs) and alkali-labile sites (ALSs) was assayed in whole blood using alkaline single-cell gel electrophoresis (SCGE; comet assay) method as described by Singh et al. [34] and modified by Mc Kelvey-Martin et al. [35]. One aliquot of whole blood was mixed with nine aliquots of RPMI-1640 medium containing 10% fetal calf serum and ten aliquots of 2% (in PBS) molten agarose type VII (Low gelling temperature). The mixture was then spread on a slide (Sigma-Aldrich) earlier covered with agarose type I (Low EEO) at 1% concentration in water and dried. The cells embedded in the agarose gel were lysed in cold lysing solution (2.5 M NaCl, 100 mM Na_2_EDTA, 10 mM Tris base, pH 10, with 1% Triton X-100 added just before use) at 4°C for at least 1 hour. Subsequently, DNA was unwound in alkaline electrophoresis buffer (1 mM Na_2_EDTA, 300 mM NaOH, pH >13) for 20 min to produce single stranded DNA and to express SSBs and ALSs and electrophoresed in the same alkaline conditions (30 min, 25 V, 300 mA, 0.93 V/cm). The gels were then neutralized by rinsing three times with 0.4 M Tris buffer (pH 7.5) and the slides were dried for storage.

In parallel analyses, oxidatively generated damage to DNA bases was identified using modified comet assay as described earlier by Collins et al. [36]. After lysis, the slides were washed three times with enzyme buffer (0.1 M KCl, 0.5 mM Na_2_EDTA, 40 mM HEPES-KOH, 0.2 mg/ml bovine serum albumin, pH 8) and incubated with formamidopyrimidine glycosylase (FPG) at 1 µg/ml in this buffer (kept at −80°C) for 30 min at 37°C. The slides were then electrophoresed and neutralized as described above. Finally, the slides were stained with 5 µg/ml DAPI and 50 cells from each slide were analyzed using an Olympus fluorescence microscope (a BX40 instrument; Olympus, Tokyo, Japan) equipped with an image analysis system (Comet IV, Perceptive Instruments, UK).

In the case of each individual, four slides were prepared simultaneously - two for assessment of DNA strand breakage and the other two, which included also the FPG treatment, for the assessment of total DNA damage (i.e. DNA strand breakage and oxidatively generated DNA damage). Relative amount of DNA in the comet tail (henceforth referred to as % DNA) obtained based on computer-aided image analysis was used as the marker of respective DNA damage. Oxidatively generated DNA damage was expressed as the difference between the total DNA damage and DNA strand breakage. For the purposes of analysis, the ratio of oxidatively generated DNA damage to DNA strand breakage was used and was calculated as



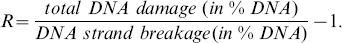



### Statistical analysis

Both absolute and relative allelic/genotypic frequencies are provided for each analyzed SNP. The Hardy-Weinberg equilibrium (HWE) was tested by a goodness-of-fit chi-square test. Inter-group comparisons of observed allelic/genotypic frequencies were performed using unconditional logistic regression and the strength of association between SNPs and breast cancer was expressed by means of age-adjusted odds ratio (OR). All ORs are provided together with corresponding 95% confidence intervals (95% CI). In the case of genotypic comparisons, both dominant and recessive genetic models were assumed.

To account for possible non-random associations between investigated *Rad51C* SNPs, linkage disequilibrium analysis and haplotype reconstruction were performed using the Haploview package [37]. LD was expressed by means of a normalized measure of allelic association |D’| [38] and LD blocks were inferred using the confidence interval method described by Gabriel et al. [39]. Briefly, a pair of SNPs is said to be in “strong LD”, if the upper confidence bound of |D’|>0.98 and the lower one >0.7. Contrary to this, a “strong recombination” between a pair of SNPs is inferred, if the upper confidence bound of |D’|<0.90. All other possibilities were classified as inconclusive. A block of “strong LD” (or a haplotype block) is defined as a region in which 95% of comparisons among informative SNPs show strong evidence of LD. Haplotypes reconstructed within each block were tested for differences in their frequencies between the control and BrC group and the significance of such differences was assessed using the two-sided exact mid-P test. Possible linkage between haplotypes and BrC was assessed based on OR with corresponding 95% CI.

Normal distribution of numerical data was tested by means of the Shapiro-Wilk *W* test. Data departing from normal distribution are provided as median [interquartile range]. As data for TBARS and relative amount of DNA in comet tails met all relevant criteria for normal distribution but were found to be significantly right-skewed, they were logarithmically transformed prior to any further analyses. Transformed data fulfilling all the criteria for normal distribution are presented as mean ± standard deviation (SD) and expressed in log-units. Analysis of outliers was performed on the basis of estimated Cook's distances. Inter-group comparisons of normally distributed data adjusted for differences in age were performed using the two-way analysis of covariance (ANCOVA) involving the subjects' age as covariate. Post-hoc comparisons were performed using the Scheffé test. In all analyses investigating the effect of *RAD51C* SNPs on markers of DNA damage, only the dominant genetic model was assumed due to limited number of rare homozygotes. In the case of blood plasma TBARS, dominant as well as additive genetic models were used. The recessive genetic model was omitted due to low number of rare homozygotes.

The significance of differences between groups in other non-paired data (such as individuals' age, sex and tobacco consumption) were assessed using the Mann-Whitney *U*, Student's *t*, or two-sided exact mid-P test, depending on the character and distribution of data. The Spearman's rank correlation (*R_S_*) was used to identify possible additional confounders, which were also involved in ANCOVA when appropriate to ensure the robustness of identified effects.

All statistical calculations were performed using the Statistica 8.0 package (StatSoft, Tulsa, USA).

## Results

### Genetic variability of *RAD51C* SNPs and association with breast cancer

Single-site analyses of all SNPs with respect to their possible association with BrC risk were performed. All analyzed SNPs presented the observed genotype frequencies which were found to be in agreement with the ones predicted by the Hardy-Weinberg law in both the control and breast cancer groups. Nevertheless, comparing the allelic frequencies between the two groups, none of the analyzed SNPs of *RAD51C* was found to present significantly different distributions of its alleles, suggesting that neither allele of any SNP can be assumed to be associated with increased BrC risk. Comparing the genotypic frequencies, neither SNP was found to present significantly different distributions of corresponding genotypes, either under dominant or recessive genetic models (Table 3).

**Table 3:** Observed frequencies of genotypes and haplotypes reconstructed based on SNPs located within the LD block identified in *RAD51C*, together with respective odds ratios (OR) and corresponding 95% confidence intervals in BrC cases and healthy controls.

SNP	BrC cases	Controls	OR [95% CI]^a^^,^ b	DNA damage control subset c
**rs302874**				
CC	55 (0.42)	69 (0.37)	A: 0.9 [0.7 – 1.3]	16 (0.40)
CT	56 (0.42)	92 (0.49)	D: 0.8 [0.5 – 1.2]	19 (0.48)
TT	21 (0.16)	27 (0.14)	R: 1.1 [0.6 – 2.1]	5 (0.13)
**rs12946522**				
TT	88 (0.67)	119 (0.64)	A: 0.8 [0.5 – 1.2]	29 (0.73)
TG	41 (0.31)	55 (0.29)	D: 0.9 [0.5 – 1.4]	8 (0.20)
GG	3 (0.02)	13 (0.07)	R: 0.5 [0.1 – 1.8]	3 (0.07)
**rs302873**				
CC	54 (0.41)	68 (0.36)	A: 0.9 [0.7 – 1.2]	15 (0.38)
CG	56 (0.42)	86 (0.46)	D: 0.8 [0.5 – 1.2]	17 (0.42)
GG	22 (0.17)	34 (0.18)	R: 0.9 [0.5 – 1.6]	8 (0.20)
**rs16943176**				
GG	86 (0.65)	118 (0.63)	A: 0.9 [0.6 – 1.3]	30 (0.74)
GA	43 (0.33)	63 (0.34)	D: 0.9 [0.6 – 1.5]	9 (0.23)
AA	3 (0.02)	7 (0.04)	R: 0.9 [0.2 – 3.7]	1 (0.03)
**rs12946397**				
CC	85 (0.64)	119 (0.63)	A: 0.9 [0.6 – 1.4]	30 (0.74)
CT	44 (0.33)	61 (0.32)	D: 1.0 [0.6 – 1.6]	9 (0.23)
TT	3 (0.02)	8 (0.04)	R: 0.7 [0.2 – 2.9]	1 (0.03)
**rs17222691**				
CC	88 (0.67)	119 (0.63)	A: 0.9 [0.6 – 1.3]	30 (0.74)
CT	41 (0.31)	62 (0.33)	D: 0.9 [0.5 – 1.4]	9 (0.23)
TT	3 (0.02)	7 (0.04)	R: 0.9 [0.2 – 3.7]	1 (0.03)
**rs28363302**				
CC	114 (0.86)	152 (0.80)	A: 0.7 [0.4 – 1.2]	32 (0.80)
C/del	17 (0.13)	36 (0.19)	D: 0.7 [0.4 – 1.4]	8 (0.20)
del/del	1 (0.01)	1 (0.01)	R: 1.3 [0.1 – 21.5]	0 (0.00)
**Haplotype**	**BrC cases**	**Controls**	**OR [95% CI] d**	
**GCC**	214 (0.811)	298 (0.793)	1.1 [0.8 – 1.7]	-
**ATT**	47 (0.178)	76 (0.202)	0.9 [0.6 – 1.3]	-
**ATC**	2 (0.008)	0 (0.000)	*NA*	-
**GTC**	1 (0.004)	1 (0.003)	1.4 [0.04 – 55.8]	-
**ACC**	0 (0.000)	1 (0.003)	*NA*	-

Data presented as absolute (relative) frequencies of individual genotypes observed in BrC cases, controls and in subjects randomized into DNA damage control subset. *NA –* not applicable.

a Genetic model employed in order to analyze the association between genotype distribution and cancer: A – direct comparison of the frequency of alleles; D – dominant genetic model; R – recessive genetic model;

b OR values adjusted for age and smoking status, together with 95% CI determined by logistic regression;

c Significant differences in genotype distributions between the whole control group and the DNA damage control subjects were sought for by the generalized Fisher exact (Fisher-Freeman-Halton) test. No significant differences were found;

d OR values and 95% CI determined by two-sided exact mid-P test.

**Figure 1:**
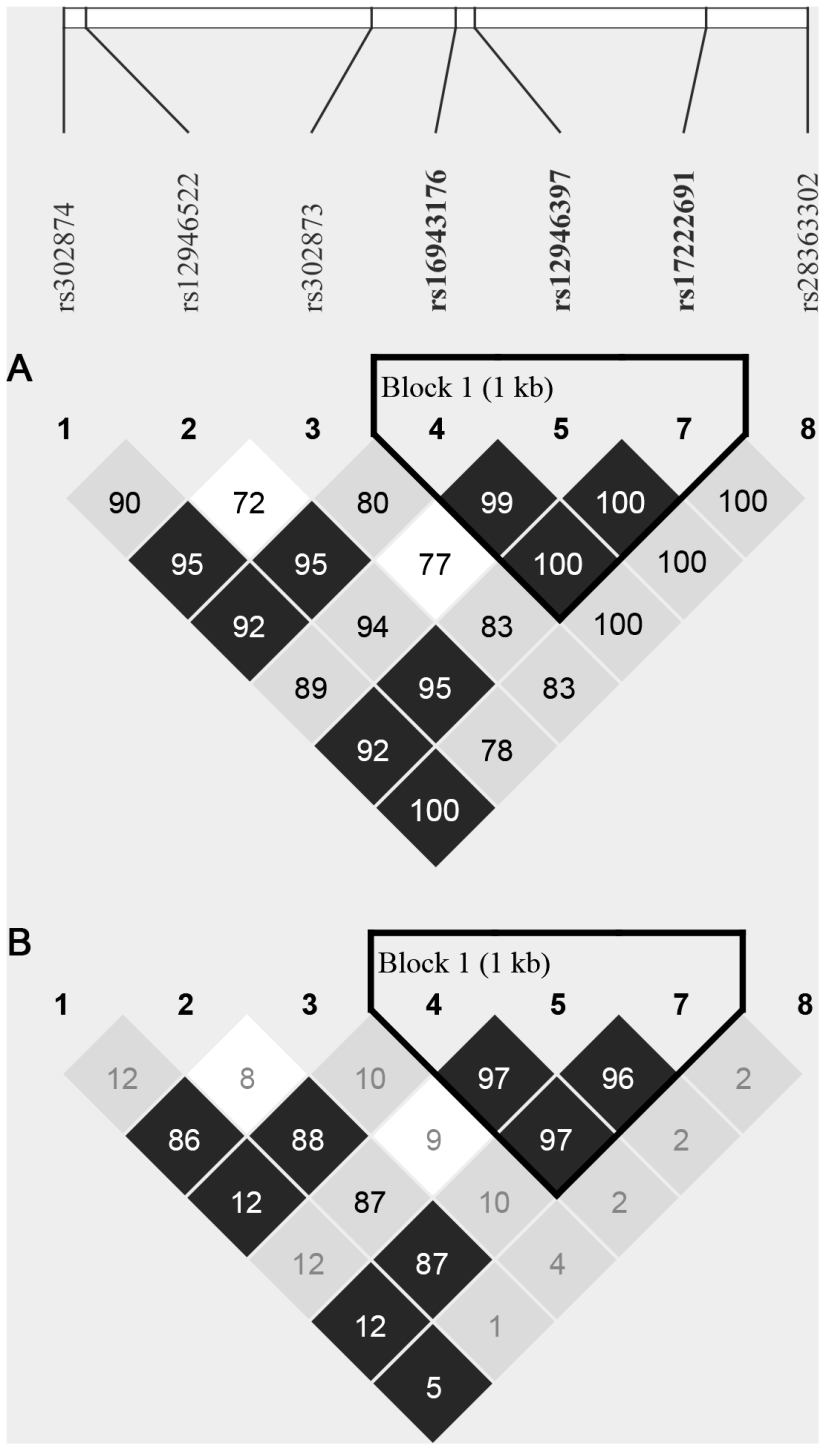
The map of LD between seven analyzed SNPs in non-coding regions of *RAD51C*. The values in the map are the normalized measures of allelic association |D’| (**A**) and correlation coefficients (r^2^) (**B**) calculated for each pair of SNPs (both provided as percentages). The color scheme represent the corresponding confidence bounds for a given pair of SNPs: black – strong evidence of LD; grey – inconclusive; white – strong evidence of recombination [37]. Solid line indicates the identified 1 kb-long LD block, within which common and rare haplotypes were reconstructed, the frequency of which is provided in the Table 3. Algorithm employed for LD block identification is described in the *Material and Methods* section.

Analysis of LD between *RAD51C* SNPs revealed a “strong LD” block spanning from rs16943176 (c.-118G>A) in 5′near gene region, through rs12946397 (c.-26C>T) in exon 1 to rs17222691 (c.145+947C>T) in intron 1 of *RAD51C* (Figure 1). Within this LD block, three rare and two common haplotypes (^−118^G/^−26^C/^145+957^C and ^−118^A/^−26^T/^145+957^T) were reconstructed. Common haplotypes encompassed together 99.7% of all haplotypes (Table 3), nevertheless, they failed to present any significant differences in distribution between controls and BrC subjects, suggesting that haplotypes in non-coding region of *RAD51C* neither are associated with the increased risk of BrC.

### Effect of *RAD51C* SNPs on blood plasma levels of TBARS. Interaction with BrC

**Figure 2:**
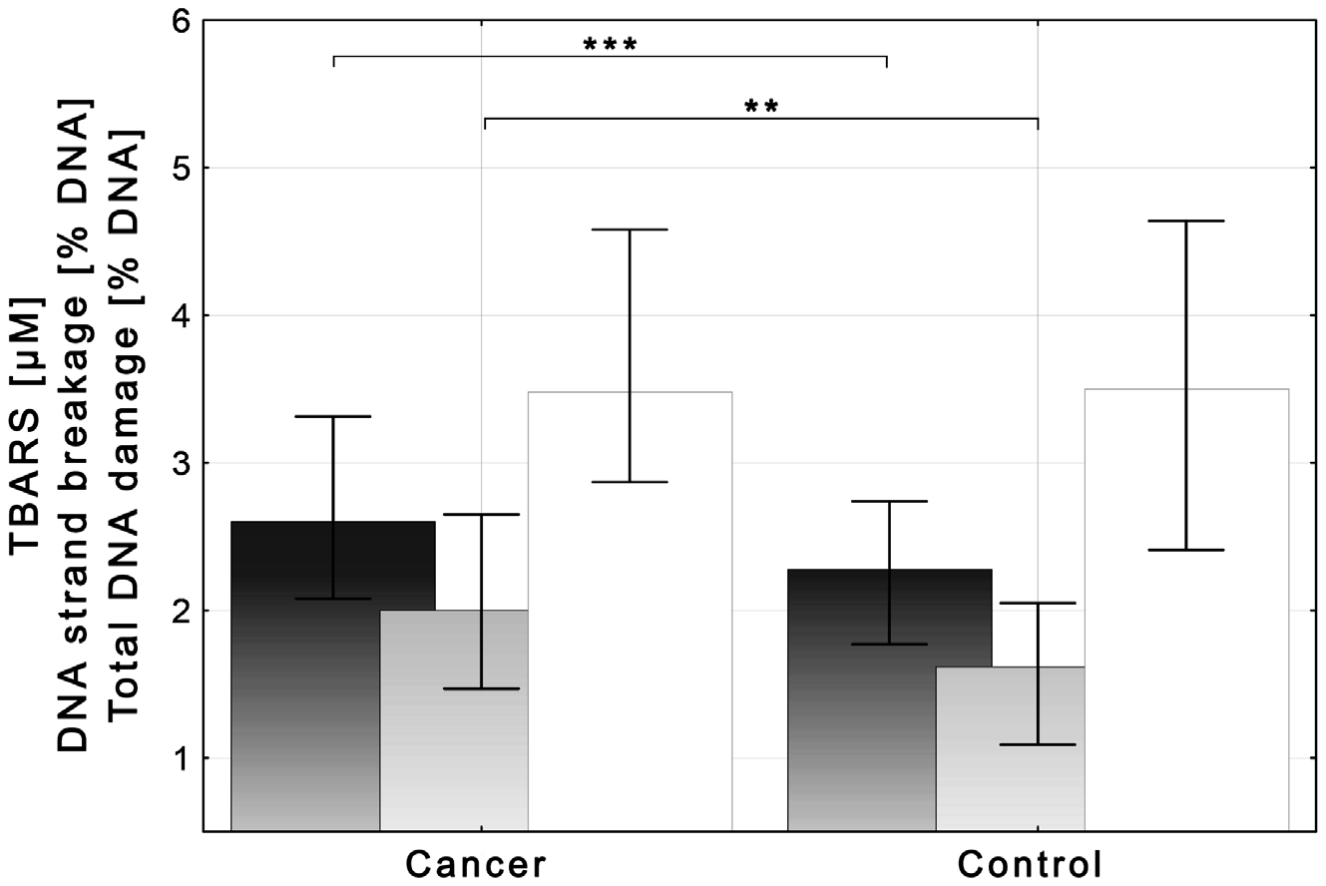
Levels of TBARS (dark columns), DNA strand breakage (grey columns) and total DNA damage (white columns) determined in whole blood samples from BrC cases and healthy control females. Data presented as medians (columns) and interquartile ranges (whiskers). Levels of TBARS among BrC cases (n = 132) vs. control females (n = 189): 2.6 [2.1–3.3] μM vs. 2.3 [1.8–2.7] μM, p<0.0001, Mann-Whitney *U* test (***). Levels of DNA strand breakage and total DNA damage were assessed in the whole group of BrC cases and tested for differences against a subset of 40 control subjects randomly selected from the whole control group by means of the age-stratified randomization (*see Materials and Methods*). DNA strand breakage: BrC cases vs. controls: 2.0 [1.5–2.7] %DNA in comet tail vs. 1.6 [1.1–2.1] %DNA in comet tail, p<0.001, Mann-Whitney *U* test (**). Total DNA damage: BrC cases vs. controls: 3.5 [2.9–4.6] %DNA in comet tail vs. 3.5 [2.4–4.6] %DNA in comet tail, *NS*. The age distribution in the control subset was verified to match with the one in the whole control group (*see* *Table 1*). The distribution of TBARS in the randomized subset of 40 controls did not differ significantly from the one observed in the whole control group (1.7 [1.5–2.1] μM vs. 2.3 [1.8–2.7] μM, *NS*) but differed significantly from the one in the BrC group (1.7 [1.5–2.1] μM vs. 2.6 [2.1–3.3] μM, p<0.001, Kruskal-Wallis *H*-test post-hoc analysis).

Overall levels of TBARS in blood plasma from BrC cases and healthy controls are shown in Figure 2, and were found to be significantly higher among BrC cases compared to controls (p<0.0001). To investigate how *RAD51C* SNPs interact with BrC in affecting the blood plasma levels of TBARS, age-adjusted single-site ANCOVA analyses, involving one *RAD51C* SNP at a time were performed. Moreover, the subjects' lifetime tobacco consumption, which was found to differ between the groups (see Table 1), correlated significantly with blood plasma TBARS (*R_S_* = 0.248; p<0.005), and was thus also involved in ANCOVA as confounder. Log-transformed levels of TBARS in blood plasma of controls and BrC cases with respect to carried genotype, under both additive and dominant genetic models, are presented in Table 4.

**Table 4:** Effect of *RAD51C* SNPs and BrC on blood plasma log-levels of TBARS.

A							
	TBARS [log-μM]						
Controls	rs302874	rs12946522	rs302873	rs16943176	rs12946397	rs17222691	rs28363302
*wt*-homozygotes	0.37±0.12	0.31±0.11	0.37±0.12	0.32±0.11	0.32±0.11	0.32±0.11	0.35±0.14
heterozygotes	0.33±0.13	0.44±0.12	0.33±0.13	0.43±0.12	0.43±0.12	0.43±0.12	0.35±0.14
*rare* homozygotes	0.36±0.15	0.29±0.04	0.36±0.14	0.27±0.13	0.27±0.13	0.27±0.13	0.54
heterozygotes & *rare* homozygotes	0.34±0.13	0.42±0.13	0.34±0.13	0.42±0.13	0.42±0.13	0.42±0.13	0.35±0.11
**BrC cases**							
*wt*-homozygotes	0.40±0.15	0.39±0.14	0.40±0.15	0.39±0.15	0.39±0.15	0.40±0.15	0.42±0.15
heterozygotes	0.42±0.17	0.44±0.18	0.41±0.16	0.44±0.17	0.44±0.17	0.43±0.17	0.38±0.12
*rare* homozygotes	0.42±0.16	0.48±0.20	0.44±0.17	0.48±0.20	0.48±0.20	0.48±0.20	0.32
heterozygotes & *rare* homozygotes	0.42±0.16	0.45±0.18	0.42±0.16	0.45±0.17	0.45±0.17	0.44±0.17	0.36±0.11

B							
	*Summary of analysis*						
**Main effect: BrC status**							
Controls	0.35±0.13						
BrC cases	0.41±0.16						
**Main effect: *RAD51C* SNP**	**rs302874**	**rs12946522 ^a^**	**rs302873**	**rs16943176 ^b^**	**rs12946397 ^b^**	**rs17222691 ^c^**	**rs28363302**
*wt*-homozygotes	0.39±0.14	0.35±0.14	0.39±0.14	0.36±0.14	0.36±0.14	0.36±0.14	0.38±0.15
heterozygotes	0.37±0.15	0.44±0.15 ^d^	0.37±0.15	0.43±0.15^d^	0.43±0.15^ d^	0.43±0.15	0.36±0.13
*rare* homozygotes	0.39±0.15	0.38±0.16	0.40±0.16	0.43±0.19	0.43±0.19	0.43±0.19	0.43±0.15
heterozygotes & *rare* homozygotes	0.38±0.15	0.43±0.15 ^e^	0.38±0.15	0.43±0.15^ e^	0.43±0.15^ e^	0.43±0.15^ f^	0.36±0.11

(**A**) Log-transformed levels of TBARS in blood plasma of healthy controls and BrC cases by carried genotype. (**B**) Summary of the analysis of main effects of *RAD51C* SNP and BrC on blood plasma levels of TBARS, adjusted to subjects' age and lifetime smoking. In each column data are presented as means ± SD. Identification of genotypes as wild-type (*wt*)-homozygotes, heterozygotes and *rare* homozygotes with reference to Table 3, in which individual genotypes for each SNP are ordered accordingly. The levels of significance of main effects and post-hoc multiple comparisons were inferred by ANCOVA and the *Scheffé* test, respectively. Main effects: ^a^ p <0.05 and p <0.01 under additive and dominant model, respectively; ^b^ p <0.05 under additive and dominant models; ^c^ p <0.05 under dominant model; Post-hoc comparisons: ^d^ p <0.05 compared to *wt-*homozygotes; ^e^ p <0.01 compared to *wt-*homozygotes; ^f^ p <0.05 compared to *wt-*homozygotes.

Age-adjusted ANCOVA involving lifetime smoking as confounder did not confirm the BrC status as the factor significantly affecting the blood plasma level of TBARS, despite the levels of TBARS in blood plasma of BrC females were slightly (but insignificantly) higher compared to the one among healthy controls (0.41±0.16 vs. 0.35±0.13 log-μM, *NS*; Table 4B).

Under additive genetic model, the main effects of rs12946522 (c.-1902T>G), rs16943176 (c.-118G>A) and rs12946397 (c.-26C>T), unlike the remaining four SNPs (i.e. rs302874 (c.-200A>G), rs302873 (c.-524C>G), rs17222691 (c.145+947C>T) and rs28363302 (c.146–706delC)), on blood plasma TBARS were all found to be statistically significant (p<0.05 for main effects of the three SNPs adjusted for age and lifetime tobacco consumption). No statistically significant interaction between the genetic variability of *RAD51C* and BrC was found, for any of investigated *RAD51C* SNPs. Post-hoc multiple comparisons showed that the significance of main effects of rs12946522, rs16943176 and rs12946397 on blood plasma level of TBARS was due to significantly higher levels of TBARS in heterozygotic females (in both BrC cases and controls taken together) compared to their respective wild-type homozygotic counterparts (^−1902^TG vs. ^−1902^TT: 0.44±0.15 vs. 0.35±0.14 log-μM, p<0.05; ^−118^GA vs. ^−118^GG, ^−26^CT vs. ^−26^CC: 0.43±0.15 vs. 0.36±0.14 log-μM, p<0.05; Table 4B). Similar effects of the three SNPs with the same direction were observed in both BrC and control groups considered separately, but the resultant differences were not statistically significant (Table 4A).

Results of analyses assuming dominant genetic model confirm those described above as statistically significant main effects of rs12946522, rs16943176, rs12946397 and additionally also the rs17222691 on blood plasma levels of TBARS were found (p<0.01 and p<0.05 for age-and-tobacco-consumption-adjusted main effects of rs12946522, and rs16943176, rs12946397, rs17222691, respectively). It turned out that the blood plasma level of TBARS in females carrying rare-allele-containing genotypes was significantly higher compared to the one of respective wild-type homozygotes (^−1902^TG&^−1902^GG vs. ^−1902^TT: 0.43±0.15 vs. 0.35±0.14 log-μM, p<0.01; ^−118^GA&^−118^AA vs. ^−118^GG and ^−26^CT&^−26^TT vs. ^−26^CC: 0.43±0.15 vs. 0.36±0.14 log-μM, p<0.01; ^145+947^CT&^145+947^TT vs. ^145+947^CC: 0.43±0.15 vs. 0.36±0.14 log-μM; p<0.05; Table 4B). Again, similar effects of the three SNPs with the same direction, although insignificant, were observed in the BrC and control subgroups (Table 4A).

### Effect of *RAD51C* SNPs on DNA damage. Interaction with BrC

**Figure 3:**
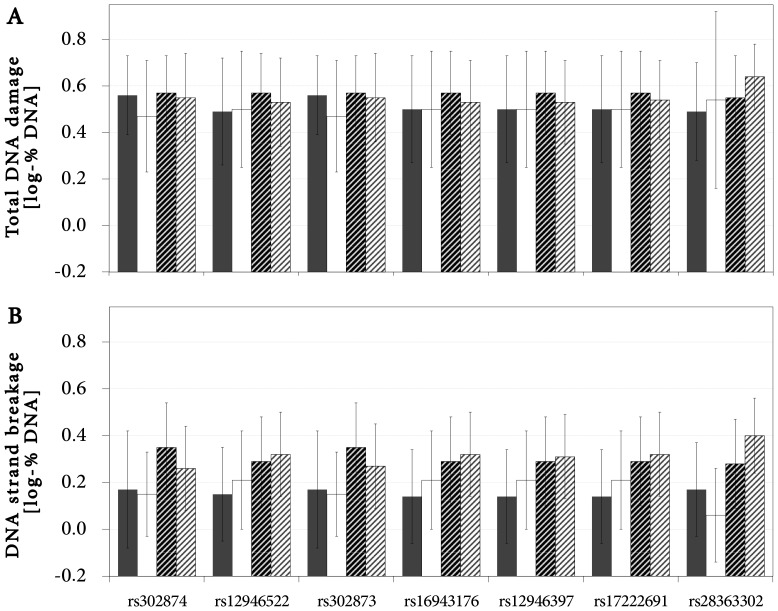
Log-transformed levels of total DNA damage (A) and DNA strand breakage (B) expressed as logarithmically-transformed relative amount of DNA in respective comet tails in whole blood leukocytes of healthy controls (solid) and BrC cases (stripes) carrying the respective wild-type (*wt-*; dark columns) or rare-allele containing (*var*-; light columns) genotypes. Only dominant genetic model was assumed. Identification of genotypes as *wild-type* or rare-allele containing (i.e. heterozygotic and *rare* homozygotes) with reference to Table 3, in which individual genotypes for each SNP are ordered accordingly. Data are presented as means (columns) ± SD (error bars). Logarithmical transformation was employed in order to normalize the distribution of raw comet assay data for the purpose of ANOVA/ANCOVA analysis. Total DNA damage data for *wt*-controls, *var*-controls, *wt-*cases and *var*-cases, respectively (log-%): **rs302874:** 0.56±0.17, 0.47±0.24, 0.57±0.16, 0.55±0.19; **rs12946522:** 0.49±0.23, 0.50±0.25, 0.57±0.17, 0.53±0.19; **rs302873:** 0.56±0.17, 0.47±0.24, 0.57±0.16, 0.55±0.19; **rs16943176:** 0.50±0.23, 0.50±0.25, 0.57±0.18, 0.53±0.18; **rs12946397:** 0.50±0.23, 0.50±0.25, 0.57±0.18, 0.53±0.18; **rs17222691:** 0.50±0.23, 0.50±0.25, 0.57±0.18, 0.54±0.17; **rs28363302:** 0.49±0.21, 0.54±0.38, 0.55±0.18, 0.64±0.14. DNA strand breakage data *wt*-controls, *var*-controls, *wt-*cases and *var*-cases: **rs302874:** 0.17±0.25, 0.15±0.18, 0.35±0.19, 0.26±0.18; **rs12946522:** 0.15±0.20, 0.21±0.21, 0.29±0.19, 0.32±0.18; **rs302873:** 0.17±0.25, 0.15±0.18, 0.35±0.19, 0.27±0.18; **rs16943176:** 0.14±0.20, 0.21±0.21, 0.29±0.19, 0.32±0.18; **rs12946397:** 0.14±0.20, 0.21±0.21, 0.29±0.19, 0.31±0.18; **rs17222691:** 0.14±0.20, 0.21±0.21, 0.29±0.19, 0.32±0.18; **rs28363302:** 0.17±0.20, 0.06±0.20, 0.28±0.19, 0.40±0.16. See Table 5 for the summary of statistical analysis.

Overall levels of DNA strand breakage and total DNA damage in the groups of BrC cases and cancer-free controls are shown in Figure 2. The level of DNA strand breakage was significantly higher in BrC cases compared to controls (p<0.001) while the total DNA damage did not vary significantly between the groups. Again, to investigate how *RAD51C* SNPs interact with BrC in affecting the total DNA damage and the DNA strand breakage, age-adjusted single-site ANCOVA analyses were performed assuming only the dominant genetic model. Since the blood plasma levels of TBARS, which differed between compared groups (see Fig. 2), correlated significantly with the level of DNA strand breakage (*R_S_* = 0.184; p<0.05), the subjects' blood plasma levels of TBARS were accounted for in ANCOVA as confounder. Log-transformed levels of total DNA damage and DNA strand breakage with respect to carried genotype are provided in Figure 3.

The analysis did not reveal the main effects of BrC as well as any of the 7 *RAD51C* SNPs on the extent of total DNA damage as being statistically significant, so did it not find any significant interaction between these two factors (Table 5A). No significant differences in the subgroups of BrC cases and controls were observable either (Figure 3). On the other hand, DNA strand breakage was found to be significantly increased among BrC cases compared to controls (p<0.005 for main effect). Nevertheless, the main effects of the 7 *RAD51C* SNPs on DNA strand breakage were not found to be statistically significant (Table 5B).

**Table 5:** Summary of the analysis of effect of *RAD51C* SNPs and BrC on DNA damage.

A	Total DNA damage [log-% DNA]						
**Main effect: BrC status**							
Controls	0.50±0.23						
BrC cases	0.56±0.18						
**Main effect: *RAD51C* SNP**	**rs302874**	**rs12946522**	**rs302873**	**rs16943176**	**rs12946397**	**rs17222691**	**rs28363302**
*wt*-homozygotes	0.57±0.16	0.56±0.19	0.57±0.16	0.56±0.19	0.56±0.19	0.55±0.19	0.54±0.19
heterozygotes & *rare* homozygotes	0.54±0.20	0.53±0.20	0.54±0.20	0.53±0.19	0.53±0.19	0.53±0.18	0.63±0.18

B	DNA strand breakage [log-% DNA]						
Main effect: BrC status ^*^							
Controls	0.15±0.20						
BrC cases	0.30±0.19 ^a^						
**Main effect: *RAD51C* SNP**	**rs302874**	**rs12946522**	**rs302873**	**rs16943176**	**rs12946397**	**rs17222691**	**rs28363302**
*wt*-homozygotes	0.33±0.20	0.26±0.20	0.32±0.21	0.26±0.20	0.26±0.20	0.26±0.20	0.26±0.20
heterozygotes & *rare* homozygotes	0.24±0.19	0.30±0.19	0.24±0.19	0.30±0.19	0.29±0.19	0.31±0.19	0.33±0.21

The summary of analysis of main effects of *RAD51C* SNPs and BrC on total DNA damage **(A)** and DNA strand breakage **(B)** adjusted to subjects' age and blood plasma TBARS. In each column, logarithmically-transformed data are presented as means ± SD. Identification of subjects as wild-type (*wt*)-homozygotes, heterozygotes and *rare* homozygotes with reference to Table 3, in which individual genotypes for each SNP are ordered accordingly. Only dominant genetic model was assumed in the analysis. The levels of significance of main effects and post-hoc multiple comparisons were inferred by ANCOVA and the *Scheffé* test, respectively. Main effects (asterisks): ^*^ p <0.01; Post-hoc comparisons (letters): ^a^ p <0.005 compared to controls.

We subsequently analyzed the effect of *RAD51C* and BrC on the ratio of oxidatively generated DNA damage to DNA strand breakage (the *R-*value). The blood plasma level of TBARS was involved in age-adjusted ANCOVA as confounder due to significant correlation with *R-*value (*R_S_* = −0.323; p<0.001). This analysis yielded the main effects of rs12946522, rs16943176, rs12946397 and rs17222691 on the ratio of oxidatively generated DNA damage to DNA strand breakage as statistically significant (age-and-TBARS-adjusted main effects: p<0.005 for rs16943176, p<0.01 in the case of rs12946522, rs12946397 and rs17222691; Table 6B). While in the carriers of respective wild-type genotypes the mean log-values of *R* ranged from −0.03 to −0.04, which corresponded to ratio of oxidatively generated DNA damage to DNA strand breakage between 0.93 and 0.91, the carriers of genotypes containing at least one respective rare allele presented significantly decreased log-values of *R* ranging from −0.28 to −0.31, corresponding to ratio of oxidatively generated DNA damage to DNA strand breakage of 0.49 to 0.52 (p<0.0005 for rs12946522 and rs16943176, p<0.001 for rs12946397 and p<0.005 for rs17222691; as compared to wild-types; Table 6B). Such effects of the four SNPs on *R*-value with the same direction were also found in the subgroups of BrC cases and healthy controls, but in the most of the cases, the resulting differences were not sufficient to prove their significance (Table 6A). No statistically significant main effects of the remaining three *RAD51C* SNPs on the ratio of oxidatively generated DNA damage to DNA strand breakage were observed. The main effect of BrC on *R-*value was found to be insignificant and so did the resultant between-group differences. No statistically significant interaction between BrC and *RAD51C* SNP influencing the *R-*value was either found.

**Table 6:** The effect of *RAD51C* SNPs and BrC on ratio of oxidatively generated DNA damage to DNA strand breakage.

A	*log-R*						
	rs302874	rs12946522	rs302873	rs16943176	rs12946397	rs17222691	rs28363302
**Controls**							
*wt*-homozygotes	0.18±0.63	0.19±0.48	0.18±0.63	0.22±0.47	0.22±0.47	0.22±0.47	0.03±0.49
heterozygotes & *rare* homozygotes	0.06±0.51	−0.19±0.63	0.06±0.51	−0.19±0.63	−0.19±0.63	−0.19±0.63	0.45±0.75
BrC cases							
*wt*-homozygotes	−0.27±0.43	−0.08±0.34	−0.25±0.43	−0.08±0.34	−0.09±0.34	−0.10±0.37	−0.17±0.41
heterozygotes & *rare* homozygotes	−0.09±0.37	−0.33±0.47 ^d^	−0.10±0.38	−0.31±0.47 ^d^	−0.30±0.47	−0.29±0.44	−0.16±0.36

B	*Summary of analysis (log-R)*						
**Main effect: BrC status**							
Controls	0.10±0.54						
BrC cases	−0.17±0.40						
**Main effect: *RAD51C* SNP**	**rs302874**	rs12946522 ^*^	**rs302873**	rs16943176 ^**^	rs12946397 ^*^	rs17222691 ^*^	**rs28363302**
*wt*-homozygotes	−0.21±0.48	−0.03±0.38	−0.19±0.48	−0.03±0.39	−0.03±0.39	−0.04±0.41	−0.13±0.43
heterozygotes & *rare* homozygotes	−0.06±0.40	−0.31±0.49 ^a^	−0.07±0.41	−0.30±0.49 ^a^	−0.28±0.49 ^b^	−0.28±0.47 ^c^	−0.04±0.50

**(A)** Values of the ratio of oxidatively generated DNA damage to DNA strand breakage *R* in whole blood leukocytes of healthy controls and BrC cases, classified by carried genotype. **(B)** Summary of the analysis of main effects of *RAD51C* SNPs and BrC on *R* ratio adjusted to subjects' age and TBARS. Provided are the logarithmically-transformed values of *R* defined as FPG[%DNA]/SSB[%DNA]-1. Data are presented as means ± SD. Identification of subjects as wild-type (*wt*)-homozygotes, heterozygotes and *rare* homozygotes with reference to Table 3, in which individual genotypes for each SNP are ordered accordingly. Only dominant genetic model was assumed in the analysis. The levels of significance of main effects and post-hoc multiple comparisons were inferred by ANCOVA (with adjustment to subjects' age and blood plasma TBARS) and the *Scheffé* test, respectively. Main effects (asterisks): ^*^ p <0.01; ^**^ p <0.005; Post-hoc comparisons (letters): ^a^ p <0.0005 compared to *wt-*homozygotes; ^b^ p <0.001 compared to *wt-*homozygotes; ^c^ p <0.005 compared to *wt-*homozygotes; ^d^ p <0.05 compared to *wt-*homozygotes.

## Discussion

Since the first study by Meindl et al. in 2010 [13], *RAD51C* has been a subject of increasing interest and several subsequent studies employing different populations have generally delivered outcomes confirming that *RAD51C* harbors mutations associated to HBOC or unselected OC [14], [18]–[22]. Despite the fact, that these mutations seem to occur with considerably lower frequency than it was originally suggested [14], [19]–[21], *RAD51C* is nowadays considered a HBOC/OC susceptibility gene [20]. Notably, all of the studies available to date seem to be in unison reporting that these mutations are however absent or at least extremely rare in hereditary BrC.

Since the previous studies were predominantly focused on missense mutations, we conducted a study in which a case-control setup was employed to evaluate the role of seven common SNPs in non-coding regions of *RAD51C* in modulation of BrC risk. Single-site analyses did not provide any evidence in favor of the hypothesis that any of investigated SNPs may be of importance with respect to BrC risk. Analysis of reconstructed haplotypes also failed to confirm that higher structures resulting from non-random associations between SNPs within this region of *RAD51C* may alter the resultant BrC risk. Although such haplotypes were found to alter the HNC risk in our previous study [30], in the context of BrC our results seem to be in line with several recent studies, in which rs12946522, rs16943176, rs12946397 and rs17222691 were studied under the case-control setup or using the *in silico* methods in relation to BrC risk, all of which failed to confirm the significantly increased frequency of these SNPs among BrC patients [14], [18], [20], [23], [25], [40], [41]. The remaining three SNPs investigated in our study (rs302874, rs302873 and rs28363302) have not yet been studied earlier in relation to BrC, but our data seem to indicate that their involvement in BrC risk modulation is rather unlikely.

Nowadays, it is widely accepted that RAD51 paralogs are involved in HR-mediated step of DSB repair occurring during the S- and G_2_-phase of the cell cycle [42], [43], recovery of stalled or broken replication fork [44] and in repair of interstrand cross-links [12]. In addition to this, a recent study has proposed that RAD51, RAD51C and XRCC3 may all play their roles in protection of mitochondrial genome against oxidative damage as well [17]. Despite the fact that we did not find any evidence confirming a direct link between genetic variability of RAD51C and BrC risk, such new and indeed intriguing notion made us to undertake further analyses aimed to find out, whether the role of RAD51C in protection against oxidative stress and oxidatively generated DNA damage can be extrapolated to nuclear DNA as well, and if so, whether it may be associated with BrC.

To our surprise, outcomes of our analyses seems to indicate that the variability in noncoding regions of *RAD51C* is linked to changes in blood plasma levels of TBARS, a widely-used marker of oxidative stress and thus that the role of this gene in certain modulation of oxidative stress in general cannot be ruled out. More precisely, we observed a blood plasma TBARS-increasing effect being linked to rare alleles of four out of seven investigated *RAD51C* SNPs (rs12946522, rs16943176, rs12946397 and rs17222691). Such effect was observed under dominant genetic model, in the case of which we obtained relatively straightforward outcomes with high power of statistical testing (reaching 90%) implying that heterozygotic and homozygotic carriers of rare alleles exhibit increased blood plasma levels of TBARS. Under additive genetic model, this effect was observed only among heterozygotic but not homozygotic carriers of rare alleles (compared to respective wild-type homozygotes), which may be due to relatively low number of rare homozygotes in some analyses: as shown in Table 3 there were only 3 homozygotic carriers of rare alleles for each of the four SNPs under discussion. Notably, such increased blood plasma level of TBARS was observed among both the controls and BrC cases, what seems to suggest that BrC and *RAD51C* genetic variability do not interact with each other in modulation of TBARS level, and thus the increased oxidative stress possibly caused by *RAD51C* genetic variability rather cannot be assumed to be linked to BrC. Nevertheless, one may speculate that the difference in blood plasma levels of TBARS between carriers of individual genotypes of rs12946522, rs16943176, rs12946397 and rs17222691 seems to be much more pronounced among control subjects than in BrC cases (see Table 4A), suggesting that the relationship between *RAD51C* SNPs and oxidative stress may differ between the groups of BrC and control subjects. This could possibly point to direct involvement of *RAD51C* in processes imposing conditions contributing to eventual BrC development, but unfortunately, our study did not provide sufficient evidence to prove significance of such interaction. Either way, further studies are required to elucidate whether such interaction is non-existent or the apparent lack of such is rather an effect of low number of rare homozygotes enrolled in our study.

Based on data obtained for biomarkers of DNA damage, our study seems to indicate that the situation concerning the effect of *RAD51C* genetic variability on DNA damage in peripheral blood leukocytes is much more complex. Generally speaking, none of the seven *RAD51C* SNPs investigated in our study were found to influence the level of total DNA damage. But intriguingly, under dominant genetic model, the contributions of oxidatively generated DNA damage and DNA strand breakage to the total pool of detected DNA damage seemed to be in function of *RAD51C* genetic variability. This hypothesis seems to be supported by the fact that the ratio of oxidatively generated DNA damage to DNA strand breakage was found to be significantly decreased among heterozygotic and homozygotic carriers of rs12946522, rs16943176, rs12946397 or rs17222691 rare alleles compared to carriers of their respective counterparts. This implies, that genetic variability at these four loci of *RAD51C* may somehow change the characteristics of observed DNA damage in whole blood leukocytes: according to observed changes in the *R-*value, the fraction of oxidatively generated DNA damage (out of the total DNA damage) dropped from some 48% among wild-types to some 34% among rare allele carriers, with this drop being statistically significant. Combined with increased blood plasma levels of TBARS observed among rare allele carriers, it all becomes even more intriguing: it seems that carriers of at least one rare allele of rs12946522, rs16943176, rs12946397 or rs17222691 present increased oxidative stress linked to increased proportion of DNA strand breakage (with relatively even distribution between oxidatively generated DNA damage and DNA strand breakage), while the respective wild-type carriers present decreased level of oxidative stress linked with DNA damage rather presenting the characteristics of oxidatively generated damage. On one hand, the link between *RAD51C* genetic variability and blood plasma levels of TBARS we observed in our study seems to indicate that this gene might indeed be involved in protection against oxidative damage, as it was suggested to be in the case of mitochondrial DNA [17]. On the other hand, simultaneous increase of the fraction of DNA strand breakage at the cost of oxidatively generated DNA damage due to genetic variability of *RAD51C* seems to support the crucial role of this gene in HR repair of DSBs, i.e. the protection of DNA against strand breakage instead. How could it be possible that increased level of oxidative stress marker is linked to decreased fraction of oxidatively generated DNA damage to the advance of DNA strand breakage? At first sight, this outcome might seem somewhat inconsistent, as one would anticipate the increased oxidative stress to come hand in hand with increased level of oxidatively damaged DNA. Nevertheless, it has earlier been suggested that direct oxidatively generated damage to DNA bases may not be the only possible mechanism by which reactive oxygen (ROS) and nitrogen (RNS) species impose their carcinogenic effect, as other alternative mechanisms have also been suggested (such as structural alterations in DNA/chromatin, decreased efficiency of DNA polymerase and DNA repair enzymes, and/or abnormal spatial configuration of DNA; see [45]–[47] for review). One can now easily envisage a situation, in which the increased amount of ROS/RNS does not directly damage DNA bases (or such damage is readily repaired), but causes structural/functional changes in DNA strand breaks-repairing enzymes (including RAD51C), which superposed to eventual suboptimal efficacy of the enzyme associated with rare allele may lead to increased levels of markers of DNA strand breakage. This would then point to superiority of the role of RAD51C in protection against DNA strand breakage over its role in protection against oxidatively generated damage. Nevertheless, a certain dose of inconsistency still remains due to contradictory associations of *RAD51C* rare alleles with the biomarkers of oxidative stress and DNA damage. Moreover, *RAD51C* rare alleles were not found to increase the extent of DNA strand breakage directly, what also seems to be inconsistent with shifted ratio of the two types of DNA damage. Either way, it thus remains to be elucidated, whether such new function of *RAD51C* proposed in the context of mitochondrial genome may be extrapolated to nuclear DNA as well.

Of importance may be the outcome, that the effect of *RAD51C* SNPs on characteristics of DNA damage in leukocytes retained its level of significance even following the adjustment to differences in blood plasma TBARS. A question however arises as to what is the possible mechanism by which the genetic variability in non-coding regions of *RAD51C* could be involved in the protection of cells against DNA strand breaks. One possible explanation could be provided by the fact, that as predicted by is-rSNP algorithm [32], the majority of SNPs investigated in our study may be considered as regulatory, i.e. they are likely to alter the binding of transcription factors (TFs) to gene's promoter with high statistical significance (see Table 2). It is thus possible, that haplotypes reconstructed based on SNPs localized within the 1kb-long strong-LD region spanning from *RAD51C* promoter to its first intron also significantly alter the binding capacity of *RAD51C* promoter towards some of crucial TFs, leading to suboptimal transcription of *RAD51C* and eventually impaired capacity of the whole HR system. According to Transfac transcription factors position weight matrix database [48], there are more than 280 different TFs, the binding of which to *RAD51C* promoter may be significantly altered by SNPs localized within the 1-kb LD block (*data not shown*). However, no studies confirming these predictions experimentally are currently available.

One has to mention, that in or study, the BrC itself was not found to be the factor linked to increased level of oxidative stress or the total DNA damage, although it was found to be associated with increased extent of DNA strand breakage. This also seems to be in contrast to finding according to which the BrC itself does not significantly change the ratio of oxidatively generated DNA damage to DNA strand breakage. It is however interesting, that any differences in blood plasma levels of TBARS and total DNA damage in leukocytes between BrC cases and controls were readily explained by adjusting the analysis to differences in subjects' age and lifetime smoking, which is in line with a considerable wealth of knowledge supporting the view of both the age and lifetime smoking as oxidative stress-increasing factors [49], [50].

Our study admittedly suffers from few limitations. One of them is undoubtedly the relatively low number of subjects enrolled, due to which the described effects of *RAD51C* SNPs on either the TBARS level or the characteristics of DNA damage were proven significant only on the level of main effects (i.e. analyzing all the subjects together irrespective of their BrC status), with respective differences in the groups of BrC cases and healthy controls analyzed separately not reaching the limit of statistical significance. Moreover, low abundance of rare alleles due to relatively small study size rendered the use of additive genetic model either impossible or leading to unreliable results. Therefore, it would definitely be of benefit to verify our outcomes in a larger study. Despite that, those SNPs for which significant associations with oxidative stress or DNA damage were found under dominant genetic model presented sufficiently high statistical power and, moreover, were proven to be not confounded by individual smoking history or the level of oxidative stress. The other limitation is comprised by significant differences in age between the BrC and control groups. According to the current theory of carcinogenesis, one would expect BrC itself to be linked with increased oxidative stress, what in fact was observed in our study. Although it is possible that certain confounding between the effects of BrC, subjects' age and their smoking statuses on blood plasma levels of TBARS and DNA damage markers might have taken place, such confounding probably did not affect the observed effect of *RAD51C* SNPs on TBARS and DNA damage, as it was identified to be independent of BrC status and robust enough to “sustain” the involvement of these confounders into the analysis.

## Conclusion

The present study showed that SNPs localized in non-coding regions of *RAD51C* do not modulate the individual susceptibility to unselected BrC. Four of them (rs12946522, rs16943176, rs12946397 and rs17222691) may however affect the level of blood plasma TBARS and change the mutual proportions of oxidatively generated DNA damage and DNA strand breakage with no effect on total amount of DNA damage: while the wild-type homozygotes seem to be linked to significantly decreased levels of TBARS and relatively even proportion between oxidatively generated DNA damage and DNA strand breakage, the respective rare allele-containing genotypes are associated with significantly increased level of blood plasma TBARS and the DNA damage characteristics being significantly shifted towards the DNA strand breakage. As predicted by the *in silico* analysis, these SNPs may disrupt the binding of well-known transcription factors, what in turn may lead to altered capacity of DNA repair pathways in which the *RAD51C-*encoded protein takes its place. Significant shift of DNA damage characteristics towards DNA strand breakage among carriers of the rare alleles suggests that in the case of nuclear DNA, the role of this protein in HR strand break DNA damage response pathway could be superior to its possible role in protection against oxidative DNA damage recently suggested in the case of mitochondrial DNA. Taken together, although we did not find any evidence confirming that noncoding SNPs of *RAD51C* modulate the risk of unselected BrC, they may be responsible for altered oxidative stress status and characteristics of DNA damage. Either way, the mechanisms behind the involvement of protein encoded by this putative suppressor gene in protection against oxidative stress and DNA damage deserve further investigation.

## References

[1] Ferlay J, Shin HR, Bray F, Forman D, Mathers C, et al (2010) Estimates of worldwide burden of cancer in 2008: GLOBOCAN 2008. Int J Cancer 127: 2893–2917. 21351269 10.1002/ijc.25516

[2] Fackenthal JD, Olopade OI (2007) Breast cancer risk associated with BRCA1 and BRCA2 in diverse populations. Nat Rev Cancer 7: 937–948. 18034184 10.1038/nrc2054

[3] Stratton MR, Rahman N (2008) The emerging landscape of breast cancer susceptibility. Nat Genet 40: 17–22. 18163131 10.1038/ng.2007.53

[4] Turnbull C, Rahman N (2008) Genetic predisposition to breast cancer: past, present, and future. Annu Rev Genomics Hum Genet 9: 321–345. 18544032 10.1146/annurev.genom.9.081307.164339

[5] Levy-Lahad E (2010) Fanconi anemia and breast cancer susceptibility meet again. Nat Genet 42: 368–369. 20428093 10.1038/ng0510-368

[6] Rahman N, Seal S, Thompson D, Kelly P, Renwick A, et al (2007) PALB2, which encodes a BRCA2-interacting protein, is a breast cancer susceptibility gene. Nat Genet 39: 165–167. 17200668 10.1038/ng1959 PMC2871593

[7] Seal S, Thompson D, Renwick A, Elliott A, Kelly P, et al (2006) Truncating mutations in the Fanconi anemia J gene BRIP1 are low-penetrance breast cancer susceptibility alleles. Nat Genet 38: 1239–1241. 17033622 10.1038/ng1902

[8] Ripperger T, Gadzicki D, Meindl A, Schlegelberger B (2009) Breast cancer susceptibility: current knowledge and implications for genetic counselling. Eur J Hum Genet 17: 722–731. 19092773 10.1038/ejhg.2008.212 PMC2947107

[9] Masson JY, Tarsounas MC, Stasiak AZ, Stasiak A, Shah R, et al (2001) Identification and purification of two distinct complexes containing the five RAD51 paralogs. Genes Dev 15: 3296–3307. 11751635 10.1101/gad.947001 PMC312846

[10] Badie S, Liao C, Thanasoula M, Barber P, Hill MA, et al (2009) RAD51C facilitates checkpoint signaling by promoting CHK2 phosphorylation. J Cell Biol 185: 587–600. 19451272 10.1083/jcb.200811079 PMC2711581

[11] Liu Y, Masson JY, Shah R, O’Regan P, West SC (2004) RAD51C is required for Holliday junction processing in mammalian cells. Science 303: 243–246. 14716019 10.1126/science.1093037

[12] Somyajit K, Subramanya S, Nagaraju G (2012) Distinct roles of FANCO/RAD51C protein in DNA damage signaling and repair: implications for Fanconi anemia and breast cancer susceptibility. J Biol Chem 287: 3366–3380. 22167183 10.1074/jbc.M111.311241 PMC3270991

[13] Meindl A, Hellebrand H, Wiek C, Erven V, Wappenschmidt B, et al (2010) Germline mutations in breast and ovarian cancer pedigrees establish RAD51C as a human cancer susceptibility gene. Nat Genet 42: 410–414. 20400964 10.1038/ng.569

[14] Vuorela M, Pylkas K, Hartikainen JM, Sundfeldt K, Lindblom A, et al (2011) Further evidence for the contribution of the RAD51C gene in hereditary breast and ovarian cancer susceptibility. Breast Cancer Res Treat 130: 1003–1010. 21750962 10.1007/s10549-011-1677-x

[15] De Leeneer K, Van Bockstal M, De Brouwer S, Swietek N, Schietecatte P, et al (2012) Evaluation of RAD51C as cancer susceptibility gene in a large breast-ovarian cancer patient population referred for genetic testing. Breast Cancer Res Treat 133: 393–398. 22370629 10.1007/s10549-012-1998-4

[16] Saleh-Gohari N, Bryant HE, Schultz N, Parker KM, Cassel TN, et al (2005) Spontaneous homologous recombination is induced by collapsed replication forks that are caused by endogenous DNA single-strand breaks. Mol Cell Biol 25: 7158–7169. 16055725 10.1128/MCB.25.16.7158-7169.2005 PMC1190269

[17] Sage JM, Gildemeister OS, Knight KL (2010) Discovery of a novel function for human Rad51: maintenance of the mitochondrial genome. J Biol Chem 285: 18984–18990. 20413593 10.1074/jbc.M109.099846 PMC2885175

[18] Osorio A, Endt D, Fernandez F, Eirich K, de la Hoya M, et al (2012) Predominance of pathogenic missense variants in the RAD51C gene occurring in breast and ovarian cancer families. Hum Mol Genet 21: 2889–2898. 22451500 10.1093/hmg/dds115

[19] Romero A, Perez-Segura P, Tosar A, Garcia-Saenz JA, Diaz-Rubio E, et al (2011) A HRM-based screening method detects RAD51C germ-line deleterious mutations in Spanish breast and ovarian cancer families. Breast Cancer Res Treat 129: 939–946. 21537932 10.1007/s10549-011-1543-x

[20] Pelttari LM, Heikkinen T, Thompson D, Kallioniemi A, Schleutker J, et al (2011) RAD51C is a susceptibility gene for ovarian cancer. Hum Mol Genet 20: 3278–3288. 21616938 10.1093/hmg/ddr229

[21] Thompson ER, Boyle SE, Johnson J, Ryland GL, Sawyer S, et al (2012) Analysis of RAD51C germline mutations in high-risk breast and ovarian cancer families and ovarian cancer patients. Hum Mutat 33: 95–99. 21990120 10.1002/humu.21625

[22] Loveday C, Turnbull C, Ruark E, Xicola RM, Ramsay E, et al (2012) Germline RAD51C mutations confer susceptibility to ovarian cancer. Nat Genet 44: 475–476. 22538716 10.1038/ng.2224

[23] Zheng Y, Zhang J, Hope K, Niu Q, Huo D, et al (2010) Screening RAD51C nucleotide alterations in patients with a family history of breast and ovarian cancer. Breast Cancer Res Treat 124: 857–861. 20697805 10.1007/s10549-010-1095-5

[24] Akbari MR, Tonin P, Foulkes WD, Ghadirian P, Tischkowitz M, et al (2010) RAD51C germline mutations in breast and ovarian cancer patients. Breast Cancer Res 12: 404. 20723205 10.1186/bcr2619 PMC2949649

[25] Wong MW, Nordfors C, Mossman D, Pecenpetelovska G, Avery-Kiejda KA, et al (2011) BRIP1, PALB2, and RAD51C mutation analysis reveals their relative importance as genetic susceptibility factors for breast cancer. Breast Cancer Res Treat 127: 853–859. 21409391 10.1007/s10549-011-1443-0

[26] Pang Z, Yao L, Zhang J, Ouyang T, Li J, et al (2011) RAD51C germline mutations in Chinese women with familial breast cancer. Breast Cancer Res Treat 129: 1019–1020. 21597919 10.1007/s10549-011-1574-3

[27] Clague J, Wilhoite G, Adamson A, Bailis A, Weitzel JN, et al (2011) RAD51C germline mutations in breast and ovarian cancer cases from high-risk families. PLoS One 6: e25632. 21980511 10.1371/journal.pone.0025632 PMC3182241

[28] Pelttari LM, Nurminen R, Gylfe A, Aaltonen LA, Schleutker J, et al (2012) Screening of Finnish RAD51C founder mutations in prostate and colorectal cancer patients. BMC Cancer 12: 552. 23176254 10.1186/1471-2407-12-552 PMC3522023

[29] Gresner P, Gromadzinska J, Polanska K, Twardowska E, Jurewicz J, et al (2012) Genetic variability of Xrcc3 and Rad51 modulates the risk of head and neck cancer. Gene 504: 166–174. 22613844 10.1016/j.gene.2012.05.030

[30] Gresner P, Gromadzinska J, Twardowska E, Rydzynski K, Wasowicz W (2014) Rad51C: A novel suppressor gene modulates the risk of head and neck cancer. Mutat Res 762: 47–54. 24631219 10.1016/j.mrfmmm.2014.02.007

[31] Sherry ST, Ward MH, Kholodov M, Baker J, Phan L, et al (2001) dbSNP: the NCBI database of genetic variation. Nucleic Acids Res 29: 308–311. 11125122 10.1093/nar/29.1.308 PMC29783

[32] Macintyre G, Bailey J, Haviv I, Kowalczyk A (2010) is-rSNP: a novel technique for in silico regulatory SNP detection. Bioinformatics 26: i524–30. 20823317 10.1093/bioinformatics/btq378 PMC2935445

[33] Wasowicz W, Nève J, Peretz A (1993) Optimized steps in fluorometric determination of thiobarbituric acid-reactive substances in serum: importance of extraction pH and influence of sample preservation and storage. Clin Chem 39: 2522–2526. 8252725

[34] Singh NP, McCoy MT, Tice RR, Schneider EL (1988) A simple technique for quantitation of low levels of DNA damage in individual cells. Exp Cell Res 175: 184–191. 3345800 10.1016/0014-4827(88)90265-0

[35] McKelvey-Martin VJ, Green MH, Schmezer P, Pool-Zobel BL, De Meo MP, et al (1993) The single cell gel electrophoresis assay (comet assay): a European review. Mutat Res 288: 47–63. 7686265 10.1016/0027-5107(93)90207-v

[36] Collins AR, Duthie SJ, Dobson VL (1993) Direct enzymic detection of endogenous oxidative base damage in human lymphocyte DNA. Carcinogenesis 14: 1733–1735. 8403192 10.1093/carcin/14.9.1733

[37] Barrett JC, Fry B, Maller J, Daly MJ (2005) Haploview: analysis and visualization of LD and haplotype maps. Bioinformatics 21: 263–265. 15297300 10.1093/bioinformatics/bth457

[38] Lewontin RC (1995) The detection of linkage disequilibrium in molecular sequence data. Genetics 140: 377–388. 7635301 10.1093/genetics/140.1.377 PMC1206563

[39] Gabriel SB, Schaffner SF, Nguyen H, Moore JM, Roy J, et al (2002) The structure of haplotype blocks in the human genome. Science 296: 2225–2229. 12029063 10.1126/science.1069424

[40] Clague J, Wilhoite G, Adamson A, Bailis A, Weitzel JN, et al (2011) RAD51C germline mutations in breast and ovarian cancer cases from high-risk families. PLoS One 6: e25632. 21980511 10.1371/journal.pone.0025632 PMC3182241

[41] De LK, Van BM, De BS, Swietek N, Schietecatte P, et al (2012) Evaluation of RAD51C as cancer susceptibility gene in a large breast-ovarian cancer patient population referred for genetic testing. Breast Cancer Res Treat 133: 393–398. 22370629 10.1007/s10549-012-1998-4

[42] Johnson RD, Jasin M (2001) Double-strand-break-induced homologous recombination in mammalian cells. Biochem Soc Trans 29: 196–201. 11356153 10.1042/0300-5127:0290196

[43] Takata M, Sasaki MS, Sonoda E, Morrison C, Hashimoto M, et al (1998) Homologous recombination and non-homologous end-joining pathways of DNA double-strand break repair have overlapping roles in the maintenance of chromosomal integrity in vertebrate cells. EMBO J 17: 5497–5508. 9736627 10.1093/emboj/17.18.5497 PMC1170875

[44] Ciccia A, Elledge SJ (2010) The DNA damage response: making it safe to play with knives. Mol Cell 40: 179–204. 20965415 10.1016/j.molcel.2010.09.019 PMC2988877

[45] Nakabeppu Y, Tsuchimoto D, Furuichi M, Sakumi K (2004) The defense mechanisms in mammalian cells against oxidative damage in nucleic acids and their involvement in the suppression of mutagenesis and cell death. Free Radic Res 38: 423–429. 15293549 10.1080/10715760410001688348

[46] Halliwell B (2007) Oxidative stress and cancer: have we moved forward? Biochem J 401: 1–11. 17150040 10.1042/BJ20061131

[47] Halliwell B (2002) Effect of diet on cancer development: is oxidative DNA damage a biomarker? Free Radic Biol Med 32: 968–974. 12008112 10.1016/s0891-5849(02)00808-0

[48] Matys V, Kel-Margoulis O V, Fricke E, Liebich I, Land S, et al (2006) TRANSFAC and its module TRANSCompel: transcriptional gene regulation in eukaryotes. Nucleic Acids Res 34: D108–10. 16381825 10.1093/nar/gkj143 PMC1347505

[49] Liochev SI (2013) Reactive oxygen species and the free radical theory of aging. Free Radic Biol Med 60: 1–4. 23434764 10.1016/j.freeradbiomed.2013.02.011

[50] Franco R, Panayiotidis MI, Kirkland SIEDJ, Mena S, Ortega A, et al (2009) Oxidative stress in environmental-induced carcinogenesis. Mutat Res Toxicol Environ Mutagen 674: 36–44. 10.1016/j.mrgentox.2008.09.017 18977455

